# Experience with Contraceptive Dosage Forms and Interest in Novel PrEP Technologies in Women

**DOI:** 10.1007/s10461-023-04072-6

**Published:** 2023-05-23

**Authors:** Claudia J. Jansen van Vuuren, Lara Lewis, Ishana Harkoo, Halima Dawood, Leila E. Mansoor

**Affiliations:** Centre for the AIDS Programme of Research in South Africa (CAPRISA), Nelson R Mandela School of Medicine, University of KwaZulu-Natal, Durban, South Africa

**Keywords:** Pre-exposure prophylaxis (PrEP), Sexual and reproductive health (SRH), PrEP preference, Prior contraceptive use, Multipurpose preventive technologies

## Abstract

New pre-exposure prophylaxis (PrEP) strategies tailored to the needs and expectations of individuals at risk of HIV acquisition are needed. In the CAPRISA 082 prospective cohort study in KwaZulu-Natal, South Africa, sexually active women aged 18 to 30 reported, through interviewer-administered questionnaires, on their prior contraceptive experience and interest in both approved and potential future PrEP dosage forms (oral PrEP, long-acting injectable PrEP, and PrEP implants) between March 2016 and February 2018. Univariable and multivariable Poisson regression models with robust standard errors were used to detect associations between women’s prior and current contraceptive use and interest in PrEP options. Of 425 women enrolled, 381 (89.6%) had used at least one modern female contraceptive method previously, with injectable depot medroxyprogesterone acetate (DMPA) being used by 79.8% (n = 339). Women were more likely to show interest in a future PrEP implant if they were currently using (aRR 2.1, CI 1.43–3.07, p = 0.0001) or had ever used (aRR 1.65, CI 1.14–2.40, p = 0.0087) a contraceptive implant, and were more likely to choose an implant as their first choice method than the implant-naïve (current users aRR 3.2, CI 1.79–5.73, p < 0.0001; “ever” users aRR 2.12, CI 1.16–3.86, p = 0.0142). Women were more interested in injectable PrEP if they had used injectable contraceptives (current users aRR 1.24, CI 1.06–1.46, p = 0.0088; “ever” users aRR 1.72, CI 1.20–2.48, p = 0.0033); and were more interested in oral PrEP if they had ever used oral contraceptives (aRR 1.3, CI 1.06–1.59, p = 0.0114). This apparent relationship between women’s contraceptive experience and their interest in novel forms of PrEP in an equivalent dosage form may play a future role in strengthening HIV prevention efforts in women at high risk of HIV acquisition.

## Introduction

The HIV epidemic continues in sub-Saharan Africa, where adolescent girls and young women account for 63% of new infections in the region [1], contributing significantly to the 1.5 million new infections globally in 2020, and presenting a significant challenge to meeting the UNAIDS 2030 goal of ending the AIDS epidemic [1, 2]. These young women bear the additional burden of high unwanted pregnancy rates resulting from the same complex sociocultural factors which increase their risk of HIV infection, such as gender inequality and limited decision-making ability regarding sexual and reproductive health [3]. Tackling new infections amongst this high-risk group requires a comprehensive approach, including improving access to effective HIV prevention technologies which suit the individual and are under their control [2–4].

In South Africa, primary health care (PHC) services are provided through a nurse-based, doctor-supported system, which include HIV testing, treatment and prevention, as well as contraception services [5]. PHC clinics offer several forms of female contraception such as injectable agents, subdermal implants, intrauterine devices and oral contraceptive pills. Oral pre-exposure prophylaxis (PrEP), specifically emtricitabine with tenofovir disoproxil fumarate (F/TDF) has been made available to people at substantial risk of HIV infection in South Africa since 2016, with subsequent updates broadening eligible groups and simplifying PrEP initiation [6, 7]. PrEP has been shown to be effective when taken daily, but poor adherence limits the efficacy of oral formulations [8–10].

To address this limitation, several novel PrEP technologies have been developed and approved. Both the dapivirine ring [11] and long-acting cabotegravir [12] have been approved for use by the South African Health Products Regulatory Authority, with yet more formulations under investigation. The dapivirine ring was shown to reduce HIV transmission by 31% in The Ring Study [13] and 27% in the ASPIRE phase 3 trial [14], where acceptability was high and most trial participants were interested in future use [15, 16]. Cabotegravir was proven to be safe and highly effective in heterosexual women in Africa in the HPTN 084 trial [17]. Doubts remain concerning the financial feasibility of this method as a public health measure, especially in low- and middle-income countries, given the high cost of the agents at present [18–20]. Further technologies, such as implants and monoclonal antibodies, are under investigation and none have been approved for use outside of clinical trials by regulatory bodies at the time of writing.

With the growing need to expand availability of these novel PrEP technologies to women at high risk of HIV acquisition, and against the backdrop of a robust contraceptive service at PHC level, we sought to describe the PrEP preferences in women at high risk of HIV acquisition in KwaZulu-Natal, South Africa, and determine whether previous experience with female contraceptive dosage forms may influence interest in novel PrEP technologies in these women.

## Methods

### Study Overview

The CAPRISA 082 study, described in detail elsewhere [10, 21], was a prospective cohort study exploring PrEP implementation, and enrolled sexually active, HIV uninfected women between the ages of 18 and 30 years old. Women were enrolled from both urban (eThekwini) and rural (Vulindlela) settings in KwaZulu-Natal, South Africa, between March 2016 and February 2018 and were followed up for a median of 10 months (interquartile range [IQR]: 7–14). All participants provided written informed consent. The study was approved by the University of KwaZulu‐Natal's Biomedical Research Ethics Committee (BE458/15).

Participants completed an interviewer-administered questionnaire on their demographic and sexual behaviour and provided details about their regular partners. The questionnaire explored the acceptability of HIV prevention options, including those which may become available in the future, including oral PrEP, long-acting injectables, and PrEP implants. The questionnaire briefly described the different dosage forms and frequency of administration of PrEP, and encouraged women to assume that all were effective in preventing HIV infection. Women in the study also reported on whether they had ever used modern female contraceptive methods, and what method they were using at the time of enrolment (if any). Participants received HIV prevention services and were offered contraception at study visits including HIV testing, risk reduction counselling, and sexually transmitted infection screening, testing and treatment. Participants were offered daily oral PrEP in the study, and were reimbursed for their time and travel costs in line with the South African Health Products Regulatory Authority (SAHPRA) reimbursement guidelines.

### Variables

#### Demographic and Social Details

Variables captured for each participant included their age, area of residence (rural or urban), their relationship type(s), recent sexual activity, frequency of condom use, source of income, partner employment status, and their level of worry about becoming infected with HIV.

#### Contraceptive Experience

Previous and current use of female contraception was elicited through questionnaire items with dichotomous “yes/no” answers, enquiring about prior and current use of each of three-monthly injectable depot medroxyprogesterone acetate (DMPA, Depo-Provera®), two-monthly injectable norethisterone enanthate (NET-EN, Nur-Isterate®), daily oral contraceptive pill, etonogestrel contraceptive implant (Implanon®), etonogestrel/ethinyl estradiol contraceptive vaginal ring (NuvaRing®). Intrauterine device use was enquired after, but not included in the analysis, because there is no equivalent dosage form of PrEP under investigation at present.

#### Interest in PrEP Methods

Dichotomous “yes/no” answers were obtained from participants exploring whether they had heard of different options used for prevention of HIV acquisition: long-acting ARV injectables; oral ARV pills; ARV implants; and vaginal rings containing ARVs. At the time of conceptualisation of the original study, there were no long-acting injectable or implantable PrEP formulations licensed for use, and so possible future options were hypothesized, which included two- and three-monthly injectables (in line with two- and three-monthly injectable contraceptives) and six-monthly implants. Participants were asked if they would be interested in using each of these methods of PrEP, should they become available, with answer options being “Yes”, “No” and “Maybe”. Lastly, participants were asked to choose one method which would be most suitable to incorporate into their lifestyle, if it became available in future.

### Statistical Analysis

Frequencies and proportions were used to summarize categorical variables. Univariable and multivariable Poisson regression models with robust standard errors were performed, adjusting for potential confounding variables in order to evaluate whether women’s contraceptive experience, and/or other variables, was associated with their interest in a PrEP product of a similar dosage form, as well as their single best method of choice. The two- and three-monthly injectable contraceptives were grouped for analysis, as were the two- and three-monthly injectable PrEP methods. These models were not performed for the PrEP vaginal ring, because there was only one user who had previous experience with the contraceptive ring; nor for oral PrEP which can be taken before and after sex, because this was not thought to be equivalent to any available contraceptive method explored in this study, including the daily oral contraceptive pill.

Variables included in the multivariable models were chosen based on available data on the topic, and included participant age group (< 25 years vs 25–30 years); site (rural vs urban); relationship type (in a casual relationship vs not); partner employment type (migrant worker vs not); whether the participant receives an income from their partner or not; recently sexually active (vaginal sex in the previous 30 days or not); condom use (sometimes or always uses condoms during sex, vs never); and being worried or not worried about being infected with HIV. Models of the probability of selecting the ARV implant as the single best method of choice were restricted to controlling only for age, site, recent sexual activity and condom use because of the smaller number of women selecting the ARV implant as their first choice. All statistical analyses were conducted in SAS version 9·4 (SAS Institute Inc., Cary, NC, USA) and used a significance level of 0.05.

## Results

The CAPRISA 082 study enrolled 425 women, all of whom answered the acceptability of HIV prevention options questionnaire as part of their study enrolment visit. The demographic characteristics of the cohort have been detailed previously [10], and are summarized in Table 1.

**Table 1:** Baseline demographic and behavioural characteristics of women enrolled in the CAPRISA 082 cohort (N = 425) and their regular partners

Baseline characteristic	% (N)
**Women**	
Age	
18–24	67.3 (286)
25–30	32.7 (139)
Level of education	
Less than secondary	31.1 (132)
Secondary or higher	68.9 (293)
Employment status	
Employed	5.9 (25)
Unemployed	94.1 (396)
Area of residence	
Urban	39.1 (166)
Rural	60.9 (259)
Age of sexual debut	
< 18	38.8 (163)
≥ 18	61.2 (257)
Lifetime number of sexual partners	
1 partner	29.4 (124)
2 partners	31.8 (134)
3 + partners	38.9 (164)
Current relationship(s)	
Stable partner	94.4 (401)
Married	1.9 (8)
Casual partner	5.2 (22)
Perceived risk of HIV	
No/low risk	41.9 (178)
Some/high risk	57.9 (246)
Initiated oral PrEP at enrolment	
Yes	61.6 (262)
No	38.4 (163)
**Regular partner**	
Age difference	
Partner < 5 years older or partner younger	73.1 (307)
Partner 5–10 years older	23.3 (98)
Partner > 10 years older	3.6 (15)
HIV status	
Infected	2.9 (12)
Uninfected	75.3 (317)
Unknown	21.9 (92)
Circumcision status	
Circumcised	57.6 (242)
Uncircumcised	36.4 (153)
Unknown	6 (25)
Employment type	
Employed as migrant worker	15.2 (64)
Other employment	46.2 (194)
Unemployed	38.6 (162)

Most women (89.6%, n = 381) had previously used a form of modern female contraception in their lifetimes, with over half (53.9%, n = 229) of women having used only one contraception method previously. The past contraceptive experience and baseline knowledge of and interest in PrEP products of women in this study is presented in Table 2.

**Table 2:** Contraceptive experience and baseline knowledge of and interest in PrEP products among women enrolled in the CAPRISA 082 cohort (N = 425)

	% (N)
Has ever used female contraceptive	
Three-monthly injectable	79.8 (339)
Two-monthly injectable	25.9 (110)
Daily contraceptive pill	10.8 (46)
Implant	16.7 (71)
Vaginal ring	0.2 (1)
None	10.4 (44)
Current contraceptive method	
Three-monthly injectable	44.7 (190)
Two-monthly injectable	8.0 (34)
Daily contraceptive pill	2.6 (11)
Implant	11.3 (48)
Intra-uterine device	0.7 (3)
None	32.5 (138)
Missing	0.2 (1)
Has heard of PrEP product	
Long-acting ARV injectables	32.0 (136)
Oral PrEP	97.2 (413)
Implants containing ARVs	12.7 (54)
Vaginal rings containing ARVs	36.7 (156)
Has an interest in PrEP product	
Three-monthly injection	52.1 (221)
Two-monthly injection	33.9 (144)
Daily oral PrEP	51.5 (219)
Pill before and after sex	29.2 (124)
Six-monthly implant	22.4 (95)
Monthly vaginal rings	13.4 (57)
Most-liked PrEP product	
Three-monthly injection	31.3 (133)
Two-monthly injection	5.4 (23)
Daily oral PrEP	37.2 (158)
Pill before and after sex	8.0 (34)
Six-monthly implant	10.6 (45)
Monthly vaginal rings	5.4 (23)
No method chosen	1.6 (7)
Missing	0.5 (2)

Most women (61.4%, n = 261) expressed interest in one or two of the PrEP methods presented, with 29.9% (n = 127) expressing interest in three or more methods, and 8.7% (n = 37) of women not reporting interest in any of the methods presented. The three-monthly injectable PrEP option and daily oral PrEP were of most interest to women in this study, and were the most common dosage forms of choice, while few women were interested in the vaginal PrEP ring.

Results from the univariable and multivariable regression suggested that women were more likely to be interested in a PrEP method if they had ever used a contraceptive method of similar dosage form (Table 3). In addition, women who were currently using an implant or injectable agent as their method of contraception were more likely to be interested in using a PrEP method of similar dosage form compared to those who were not currently using these methods, but this relationship was not demonstrated for those currently using the oral contraceptive pill (Table 3).

**Table 3:** Association between contraceptive use and interest in PrEP product of similar dosage form

Contraceptive form	Use of contraceptive form		Interest in PrEP method of similar dosage form, % (n/N)	Risk ratio	95% CI	p value	Adjusted risk ratio*	95% CI	p value
Injectable	Ever used	Yes	63.5 (233/367)	1.75	1.24–2.49	0.0017	1.72	1.2–2.48	0.0033
		No	36.2 (21/58)	1			1		
	Current use	Yes	65.2 (146/224)	1.21	1.03–1.42	0.018	1.24	1.06–1.46	0.0088
		No	53.7 (108/201)	1			1		
Pill	Ever used	Yes	65.2 (30/46)	1.31	1.04–1.65	0.0246	1.3	1.06–1.59	0.0114
		No	49.9 (189/379)	1			1		
	Current use	Yes	45.5 (5/11)	0.88	0.46–1.69	0.7	1.04	0.63–1.71	0.8921
		No	51.7 (214/414)	1			1		
Implant	Ever used	Yes	33.8 (24/71)	1.69	1.15–2.48	0.0081	1.65	1.14–2.4	0.0087
		No	20.1 (71/354)	1			1		
	Current use	Yes	43.8 (21/48)	2.23	1.52–3.26	< 0.0001	2.1	1.43–3.07	0.0001
		No	19.6 (74/377)	1			1		

*Adjustment variables: age group; site; relationship type; partner employment type; whether the participant receives an income from their partner or not; recently sexually active; condom use; degree of worry about being infected with HIV. Models of the probability of selecting the ARV implant as the single best method of choice were restricted to controlling only for age, site, recent sexual activity and condom use because of the smaller number of women selecting the ARV implant as their first choice

Women were more than twice as likely to select a PrEP implant as their first choice of PrEP method, were it to become available in future, if they had ever used (or were currently using) a contraceptive implant (Table 4). Women who were currently using an injectable contraceptive method were also more likely to choose an injectable PrEP method as their method of choice compared to those who were not currently using injectable contraceptives (Table 4).

**Table 4:** Association between contraceptive use of and single best choice of PrEP product of similar dosage form

Contraceptive form	Use of contraceptive form		Selected PrEP method of similar dosage form as method of choice, % (n/N)	Risk ratio	95% CI	p value	Adjusted risk ratio*	95% CI	p value
Injectable	Ever used	Yes	38.4 (141/367)	1.49	0.94–2.34	0.0879	1.52	0.96–2.4	0.0765
		No	25.9 (15/58)	1			1		
	Current use	Yes	43.8 (98/224)	1.52	1.17–1.97	0.0019	1.67	1.28–2.18	0.0001
		No	28.9 (58/201)	1			1		
Pill	Ever used	Yes	37 (17/46)	0.99	0.67–1.48	0.974	0.93	0.67–1.29	0.6634
		No	37.2 (141/379)	1			1		
	Current use	Yes	27.3 (3/11)	0.73	0.28–1.93	0.5233	0.79	0.42–1.5	0.475
		No	37.4 (155/414)	1			1		
Implant	Ever used	Yes	18.3 (13/71)	2.03	1.12–3.66	0.0195	2.12	1.16–3.86	0.0142
		No	9 (32/354)	1			1		
	Current use	Yes	27.1 (13/48)	3.19	1.8–5.64	< 0.0001	3.2	1.79–5.73	< 0.0001
		No	8.5 (32/377)	1			1		

*Adjustment variables: age group; site; relationship type; partner employment type; whether the participant receives an income from their partner or not; recently sexually active; condom use; degree of worry about being infected with HIV. Models of the probability of selecting the ARV implant as the single best method of choice were restricted to controlling only for age, site, recent sexual activity and condom use because of the smaller number of women selecting the ARV implant as their first choice

## Discussion

In this study we explored contraceptive experience and PrEP interests of women at high risk of HIV acquisition in KwaZulu-Natal, South Africa.

Reassuringly, and in contrast to previous studies such as the FEM-PrEP trial [22], an overwhelming majority of study participants reported prior (and current) use of a modern form of female contraception, with injectables and implants being used by most women.

Our findings provide evidence that there is a relationship between women’s prior or current use of contraception and their interest in equivalent dosage forms of PrEP, which may facilitate identification of women who may be interested in novel forms of PrEP in the future. Uptake of contraceptive methods has been shown to be influenced by knowledge of those methods [23]—knowledge of contraceptive methods could theoretically translate into increased uptake of equivalent dosage forms of PrEP; although PrEP acceptance and adherence has been shown to be a complex construct in women [24]. Contraceptive counselling encounters have nonetheless been recognized as important opportunities for PrEP counselling in adolescent girls and young women (AGYW) with diverse risk-reduction needs [25].

Sexual HIV transmission and unwanted pregnancies have shared risk factors and may occur in the very same women [3]; but while these groups may recognize the risk of unwanted pregnancy and importance of contraception [26], they commonly misidentify themselves as being at low risk of HIV infection [24, 27–29]. Adopting a combined approach to family planning and HIV prevention in these women, by leveraging women’s self-identified need for contraception as an opportunity to provide PrEP to them in a similar, acceptable dosage form could integrate the three recognized components of the HIV prevention cascade [30]: (1) improving risk perception and acceptability of PrEP by standardizing the discussion about the hand-in-hand nature of HIV risk and the risk of unwanted pregnancy; (2) making PrEP more accessible and available, if its provision becomes a standard approach, rather than targeting “high-risk” groups; and (3) supporting adherence to PrEP by combining PrEP provision and family planning, which women are less likely to forego.

Provision of a combination service or product may be especially beneficial in the context of community-based services [31], which remove some of the barriers AGYW face in accessing sexual and reproductive health services at clinics [26, 32], and are convenient and confidential [33]; with community-based services already having been shown to improve HIV testing services (HTS) when integrated with pre-existing family planning services [34], and facilitating PrEP uptake following home-based HTS [35].

The provision of such a service could be made more efficient in future with the development of Multipurpose Prevention Technologies (MPTs), which will allow women to meet their simultaneous needs for reliable contraception and HIV prevention with the use of a single, combination product in a dosage form most acceptable to them [36]. Several MPTs across a range of different dosage forms are currently under investigation, with the Dual Prevention Pill in the most advanced stages of development [37].

The findings of this study have limitations: the CAPRISA 082 study was exploring uptake of oral PrEP, which may have influenced interest expressed in oral PrEP. The data were collected some years ago, at a time when oral PrEP had only recently become available to the population studied, and whose knowledge and exposure to which may have been limited. Expressing interest in a product may not necessarily imply a willingness to take that product or translate to effective adherence to a product, and is but one of many socio-ecological factors that need to be considered that affect PrEP uptake and adherence, with others existing at the household, organizational, and community levels [24].

## Conclusion

In women participating in the CAPRISA 082 study, there was a relationship between interest in different dosage forms of PrEP, and prior or current use of contraception in an equivalent dosage form. Contraceptive experience may play a role in women’s future uptake of novel PrEP methods, which may be of programmatic significance as these methods become widely available in future. Novel PrEP strategies are needed to expand HIV prevention options and to meet the individual needs and expectations of women at high risk of HIV acquisition. As injectable PrEP becomes more widely available to AGYW in Africa, research should focus on the relationships between contraceptive use and adherence to injectable PrEP.

## Data Availability

Data are available from the corresponding author on reasonable request.
